# Use of Single-Frequency Impedance Spectroscopy to Characterize the Growth Dynamics of Biofilm Formation in *Pseudomonas aeruginosa*

**DOI:** 10.1038/s41598-017-05273-5

**Published:** 2017-07-12

**Authors:** Jozef B. J. H. van Duuren, Mathias Müsken, Bianka Karge, Jürgen Tomasch, Christoph Wittmann, Susanne Häussler, Mark Brönstrup

**Affiliations:** 1 Department of Chemical Biology, Helmholtz Centre for Infection Research, Braunschweig, Germany; 2 Institute of Systems Biotechnology, Saarland University, Saarbrücken, Germany; 3 Department of Molecular Bacteriology, Helmholtz Centre for Infection Research, Braunschweig, Germany; 4 Institute for Molecular Bacteriology, TWINCORE, Centre for Experimental and Clinical Infection Research, Hannover, Germany; 5 Group Microbial Communication, Helmholtz Centre for Infection Research, Braunschweig, Germany; 6 German Centre for Infection Research (DZIF), Site Hannover Braunschweig, Braunschweig, Germany

## Abstract

Impedance spectroscopy has been applied in prokaryotic and eukaryotic cytometry as a label-free method for the investigation of adherent cells. In this paper, its use for characterizing the growth dynamics of *P. aeruginosa* biofilms is described and compared to crystal violet staining and confocal microscopy. The method allows monitoring the growth of biofilm-forming *P. aeruginosa* in a continuous and label-free manner over a period of 72 h in a 96 well plate format. Impedance curves obtained for *P. aeruginosa* PA14 wild type and mutant strains with a transposon insertion in *pqsA* and *pelA* genes exhibited distinct phases. We propose that the slope of the declining curve following a maximum at ca. 35–40 h is a measure of biofilm formation. Transplant experiments with *P. aeruginosa* biofilms and paraffin suggest that the impedance also reflects pellicle formation at the liquid-air interface, a barely considered contributor to impedance. Finally, the impairment of biofilm formation upon treatment of cultures with L-arginine and with ciprofloxacin, tobramycin and meropenem was studied by single frequency impedance spectroscopy. We suggest that these findings qualify impedance spectroscopy as an additional technique to characterize biofilm formation and its modulation by small molecule drugs.

## Introduction

Infections caused by pathogenic bacteria represent a major health threat that is expected to rise further in the future^1^. The formation of biofilms is in many cases a prerequisite for the development of chronic bacterial infections that can persist for months or years^2^, because they increase the resistance of bacterial pathogens to drug therapies^3–8^. Biofilms not only constitute a physical barrier that impedes the penetration of antibiotics, but also exhibit an altered metabolism compared to planktonic bacteria^9^. In a biofilm, microbes are embedded in a self-produced matrix of extracellular polymeric substances (EPS), which consist of DNA, proteins and polysaccharides.

*P. aeruginosa* forms various types of biofilms, which differ in the composition of their EPS, depending on its genetic background and environmental cues^10^. Important polysaccharides for the three-dimensional biofilm are pel, psl and alginate^10–15^. In case of the commonly used laboratory strain PA14, the polysaccharide pel is mandatory for biofilm formation^14, 16^. Concordant, the *pelA::Tn* mutant is not capable of forming a pellicle biofilm at the air liquid interface. It is also known that the *P. aeruginosa* quinolone signal (PQS) plays a significant role as a signal molecule for the PQS quorum sensing system. The latter system regulates the production of extracellular DNA, an important biofilm matrix component^11^. Accordingly, a *psqA::Tn* mutant was found to form less biofilm in a microscopic biofilm screen as well as in a murine animal model^17–19^.

Biofilm structures are induced by nutrition cues and/or non-lethal concentrations of antibiotics^20^. *Pseudomonas aeruginosa* has become an important model organism to study biofilms, as this opportunistic Gram-negative pathogen is the frequent cause of devastating chronic, biofilm-associated infections, for example in cystic fibrosis or wound patients^21, 22^.

To develop new therapeutic solutions for such infections, there is a high demand for diagnostic methods that also characterize the biofilm phenotype of pathogens. Common techniques mostly rely on colorimetric measurements of biofilm formation^23–36^. A frequently applied method is the staining of biofilms by crystal violet; however, this technique suffers from high standard deviations, cannot determine viability and is an endpoint measurement, reflecting only a single time point. Two recent, commercially available techniques are the Calgary Biofilm Device and the Biofilm Ring Test. Both of them define antimicrobial efficacies in relation to biofilm formation in a 96 well-plate format^30, 37^. Detailed three-dimensional information on biofilms of *P. aeruginosa* was obtained by automated confocal laser scanning microscopy (CLSM) following a live and death staining of cells^33^. This 96 well-plate-based optical method could determine antibiotic efficacy in biofilms in a quantitative, spatially resolved manner. Its utility was also proven in a genetic screen for mutants affecting the formation of biofilms^18^ that allowed deducing specific biofilm phenotypes.

‘Single-frequency impedance analysis’ measures the opposition that an electrochemical system exhibits upon application of an alternating voltage. Also biological matrices constitute such an electrochemical system, featured by energy dissipater (resistor) and energy storage (capacitor) elements. The use of EIS to characterize changes in the density and morphology of eukaryotic cells adherent to an electrode surface has been pioneered by Giaever and Keese^38–40^. As the method provides a label free, non-invasive and quantitative assessment of cellular processes like protrusion, proliferation, viability, migration, trans-endothelial invasion, wound healing, apoptosis, cytotoxicity, or cell-cell adhesion, and as it is simple, prone to miniaturization and to low-cost formats, EIS has found widespread applications in cell biology, bioanalysis, and medicine^40–51^. Impedance spectroscopy was also applied since the 1970’s^52^ for the detection of bacteria in food and manufacturing industries^53^. A variety of impedance studies on bacterial biofilm formation have been reported^54–56^. Among those, Paredes *et al*. characterized the biofilm formation of *Staphylococcus epidermis* in four different devices^57^. It was shown that the change in impedance during biofilm formation in an unstirred device with a sensor on the bottom was primarily driven by the capacitance. This effect was explained by an increased charge transfer resistance by the coating of the sensor material. Gottschamel *et al*. found that contactless bioimpedance measurements are particularly sensitive toward capacitive changes during biofilm formation and maturation^58^. Kim *et al*. observed a decrease in the double-layer capacitance during adhesion and biofilm maturation of *P. aeruginosa* at the electrode surface, and speculated that bacteria obstructed double-layer charging by blocking the surface^59^. A microfluidic sensor platform was constructed by Bruchmann *et al*. to investigate the growth and the viability *of P. aeruginosa* strains in a time-dependent manner^60^. The impact of disinfectants and detergents on the mostly continuously increasing impedance curves (at a starting OD of 0.5) was also reported on that system. Ward *et al*. constructed a low-cost printed carbon sensor and used frequency sweeps at distinct time points to reveal a distinct impedance signature of *P. aeruginosa* that is shaped by the production of phenazines^61^. The authors summarize that the impedance signal is influenced by six mechanisms: (i) the production of redox compounds; (ii) the deposition of biofilm material on the electrode surface; (iii) charge transfer through the attachment of cells and microbial nanowires; (iv) the presence of microbial cells in close proximity to the electrode surface; (v) breakdown of nutrients within the electrolyte; and (vi) the adsorption of proteins to the electrode surface. Of course, the relative contributions of these mechanisms to impedance may change over the growth period of the microorganism.

In this paper, we highlight differences in the dynamics of biofilm formation of three *P. aeruginosa* strains by following the single frequency impedance spectroscopy over 72 h in a label-free manner. The setup was successfully applied to study the effects of three standard antibiotics on biofilm formation over time. Finally, we describe another contributing factor to impedance that, to the best of our knowledge, has not been considered so far, i.e. the pellicle biofilm that forms at the air-liquid interface.

All studies were performed by single-frequency impedance spectroscopy using a commercial instrument, the xCELLigence Real Time Cell Analyzer (RTCA) from Acea Biosciences that measures impedance in 96 well plates equipped with gold microelectrodes. The instrument expresses impedance in form of a standardized, dimensionless parameter termed cell-index. We suggest that the slope of the cell index at specific detection time periods can characterize pellicle biofilm formation with performance features (standard deviations and effect sizes) superior to crystal violet staining. A part of the results has been included in a patent application by us (see conflict of interest statement)^62^. Due to the automated setup using microtiter plates, impedance spectroscopy may have additional applications, e.g. for the screening of compound libraries to discover inhibitors and/or enhancers of biofilm formation.

## Results

### Characterization of *Pseudomonas aeruginosa* biofilms by inverted confocal microscopy, crystal violet staining and single-frequency impedance spectroscopy

**Figure 1:**
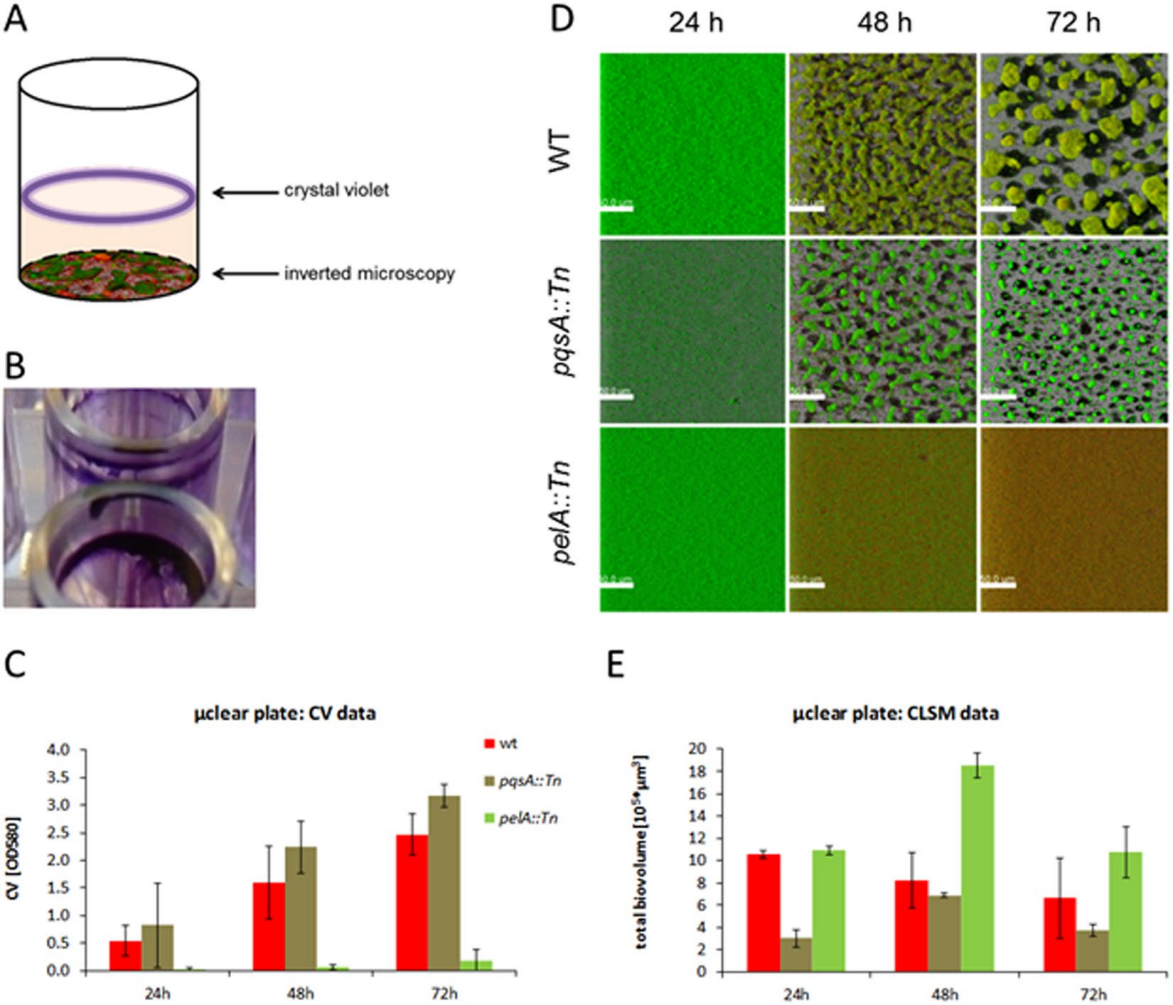
Comparison of biofilms measured with inverted confocal laser scanning microscopy (CLSM) and with crystal violet staining. (**A**) Schematic visualization of biofilm localization in a microtiter plate well. (**B**) Example of the biofilm stained with crystal violet in an E-plate 96 (ACEA, USA). (**C**) Biofilm biomass determined with crystal violet staining in a half-area, µclear microtiter plate (n = 12) (Greiner Bio-One). The crystal violet background of the medium was subtracted. (**D**) Easy 3D projections of biofilm structures stained with the BacLight Viability Kit. (**E**) Biofilm biovolume determined from biofilm structures as depicted in Fig. 1D with a customized solution of the image analysis software Definiens (n = 6). Red bars: PA14 Wild type; olive bars: *pqsA::Tn* mutant; light green bars: *pelA::Tn* mutant. n indicates the number of technical replicates.

Three different strains of *Pseudomonas aeruginosa* that were well-characterized with respect to their biofilm-forming ability, i.e. the PA14 wild type (wt) and the *pelA::Tn* and *pqsA::Tn* transposon mutants, were selected for analysis by single-frequency impedance spectroscopy. The results were compared to those obtained by confocal microscopy and crystal violet staining as reference techniques. In crystal violet staining experiments, the biofilm was visible as a ring at the air-liquid interface (pellicle), while confocal microscopy measured structures on the bottom of microtiter plates at the solid-liquid interphase (Fig. 1A,B). The pellicle staining was robust, tolerating rigorous washing steps. In contrast, the solid-liquid biofilm was very fragile and strongly dependent on the formed pellicle as well as on nutrient and oxygen levels. The pellicle biofilm of PA14 was increasingly formed over a period of 72 h in half-area µclear 96 well plates as well as in the 96 well E-plates that were used for impedance measurements (Fig. 1B,C). The *pelA::Tn* mutant did not form any pellicle in both plate types, confirming preceding reports in similar experimental setups. In contrast, the staining of the *pqsA::Tn* mutant’s pellicle was even stronger than that of the wild type. The data obtained by inverted confocal microscopy at the solid-liquid interface gave a different picture (Fig. 1D,E). Here, the *pqsA::Tn* mutant showed reduced levels of biofilm, as the calculated biovolume of the 3D biofilm stack (E) was lower compared to the wild type. In contrast, the calculated biovolumes of the *pelA::Tn* mutant were as high as (24 h, 72 h) or higher than (48 h) those of the wild type. However, the microscopic images in Fig. 1D show that the *pelA::Tn* mutant did not form a three-dimensional biofilm structure, as opposed to the wild type and the *pqsA::Tn* mutant. Visual microscopic observations revealed that the *pelA::Tn* biovolume was a result of a massive accumulation of planktonic cells, which biased the biovolume calculation. This was also visible in the time series presented and holds true for the 24 h values of the wild type and in part the *pqsA::Tn* mutant. Microtiter plate biofilms usually change over time due to changes in nutrient and oxygen availability. While the induced dispersal can be seen for the wild type and *pqsA::Tn* mutant, the appearance of *pelA::Tn* mutant was not changed. Based on crystal violet staining, the *pelA::Tn* mutant was impaired in biofilm formation at the air-liquid interface, while inverted confocal microscopy showed a reduction in biovolume for the *pqsA::Tn* mutant at the solid-liquid interface.

**Figure 2:**
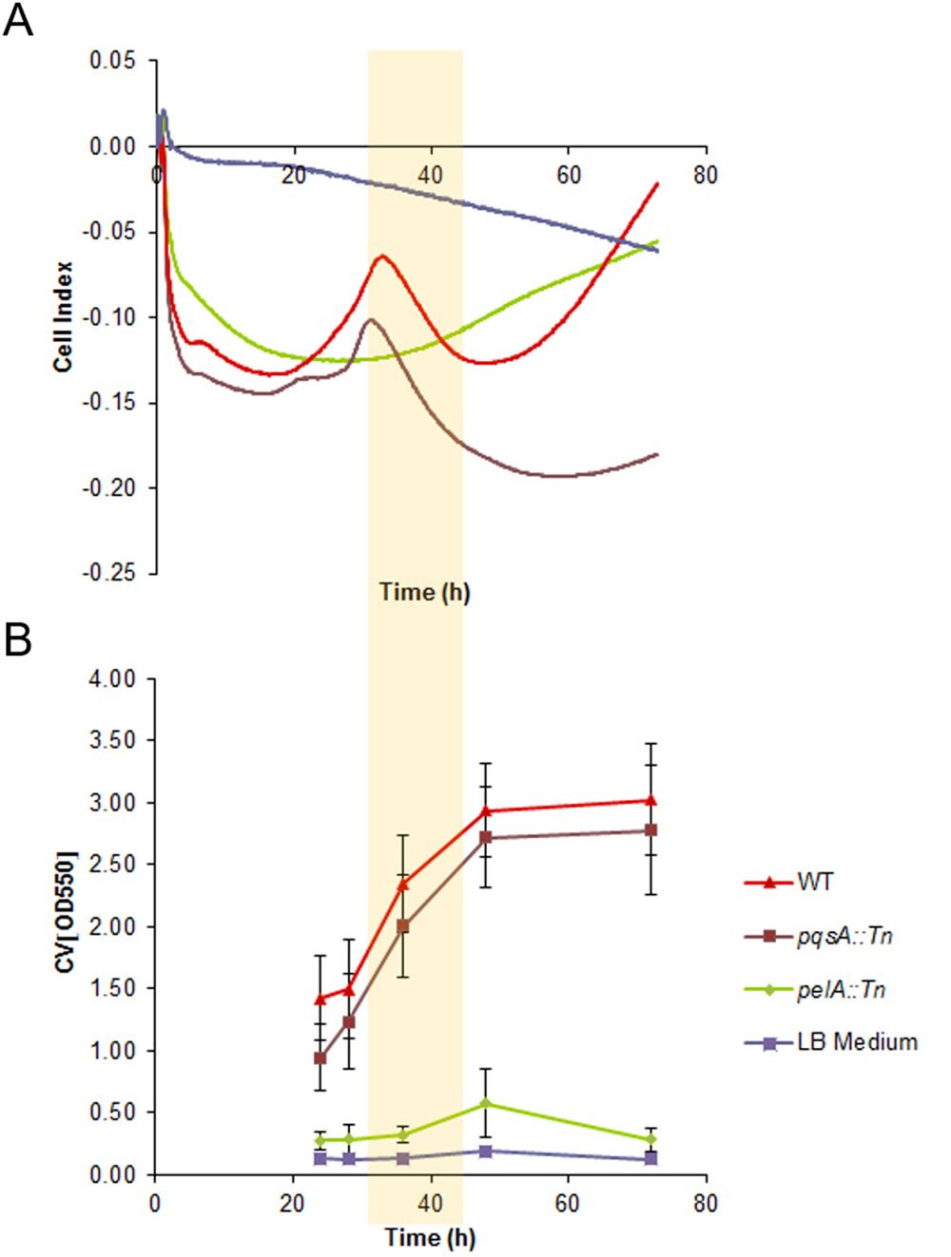
(**A**) Changes in the standardized single-frequency impedance (expressed as cell indices) and (**B**) crystal violet staining measurements of pellicle biofilm over 72 h, during biofilm growth in LB culture medium of PA14 wt, *pqsA::Tn* mutant, and *pelA::Tn* mutant (n = 24 for each mutant) and LB Medium (n = 8). The PA14 wild type and *pqsA::Tn* are biofilm formers; *pelA::Tn* does not form biofilm. n indicates the number of technical replicates. The period of linear decline of the cell index is highlighted by a yellow bar.

As most studies of impedance spectroscopy measure biofilms at distinct time points, we wanted to investigate whether the technique could capture the dynamics of biofilm formation through continuous measurements. In addition, the ability of the technique to discern differences between the strains was probed. The cell index values of the single-frequency impedance measurements over 72 h are shown in Fig. 2A. The cell index of the negative control (LB medium alone) was not constant, but decreased slowly over time. In all experiments with *P. aeruginosa*, a pronounced decay of the cell index over approximately 8 hours occurred, whose magnitude was variable between experiments. For the *pelA::Tn* mutant, a structureless, constantly increasing cell index was observed after that time period. In contrast, the curves obtained for the PA14 wt and the *pqsA::Tn* mutants increased to a maximum after ca. 33 h, and then showed a linear decay over time for a period of ca. 10 h. After a second minimum, a signal increase was observed that lasted to the end of the measurement time (see also Table S1 and Figures S2–S4). All three strains produced an approximately equal number of colony forming units per well after 72 h, demonstrating that the overall growth was equal. This finding indicates that the differences in single-frequency impedance curves did not reflect differences in growth, and that bacteria were alive after a 72 h cultivation period in the 96 well E-plate without further nutrient additions. The impedance dynamics were referenced to crystal violet staining intensities of the strains under equivalent conditions (Fig. 2B). Crystal violet staining demonstrated that the majority of the pellicle biofilm was formed in the time between 24 and 48 h. Shorter time points (<20 h) could not be captured by crystal violet, as a visible, physical biofilm was not formed in that period. Thus, in the period of biofilm formation (ca. 24–48 h), the cell index curves were featured by a local maximum and a linear decline (Fig. 2A).

### Single-frequency impedance spectroscopy detects biofilm changes at the air-liquid interface

**Figure 3:**
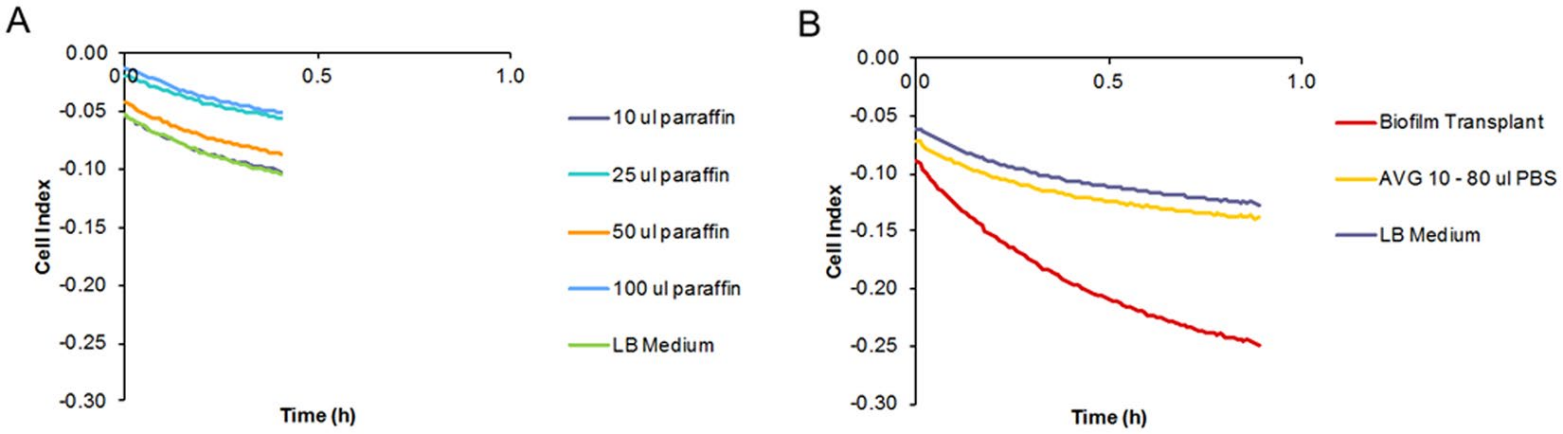
Effect of the addition of biofilm and control substances on top of the bacterial broth on single-frequency impedance. (**A**) Impedance curves following addition of paraffin in different amounts (n = 4). (**B**) Impedance curves following addition of PA14 pellicle biofilm (n = 4) or different amounts of PBS buffer (n = 1). As the curves for PBS buffer additions had identical shapes, an average curve is shown. n indicates the number of technical replicates.

In order to probe the specific effect of the pellicle biofilm on the cell index, we performed two experiments: First, we added paraffin (10–100 µl) as an artificial substrate to LB medium (Fig. 3A). Paraffin was chosen to mimic the addition of an inert, liquid material on the top of the broth solution, as it is immiscible with water and therefore floats on top of the medium due to its lower density. Because its chemical composition consists of alkanes, paraffin has a low dielectric constant, in contrast to the pellicle biofilm of *P. aeruginosa*. Secondly, a pellicle biofilm was transplanted with a metal loop into wells filled with LB medium (Figs 3B and S6). The pellicle biofilm was obtained by growing PA14 in a 96 well plate in LB medium for 72 h. We observed that the cell index was reduced by the addition of pellicle biofilm (Fig. 3B); the decrease was steeper than that obtained for the control (added PBS buffer in varying amounts of 10–80 µl). This effect cannot be explained by the addition of an immiscible layer on top of the LB medium, as the addition of paraffin led to unchanged slopes of cell index (Fig. 3A). We concluded from this experiment that the electrical single frequency impedance is strongly influenced by a bacterial biofilm at the air-liquid interface. In addition, we concluded that the declining cell index curve is a characteristic of pellicle biofilm formation.

### Pharmacological interference of biofilm formation can be monitored by single-frequency impedance spectroscopy

The cell index curves depicted in Fig. 2 demonstrate that the growth of biofilm is a process with distinct phases. We next wanted to probe whether cellular single-frequency impedance spectroscopy can capture the effects of low molecular weight compounds that interfere with biofilm formation. First, the effect was tested through the addition of 0.4% and 0.8% (w/w) L-arginine. L-Arginine is a model compound for biofilm suppression that acts by stimulating anaerobic metabolism in *P. aeruginosa* and other biofilm-forming bacteria^63, 64^.

**Figure 4:**
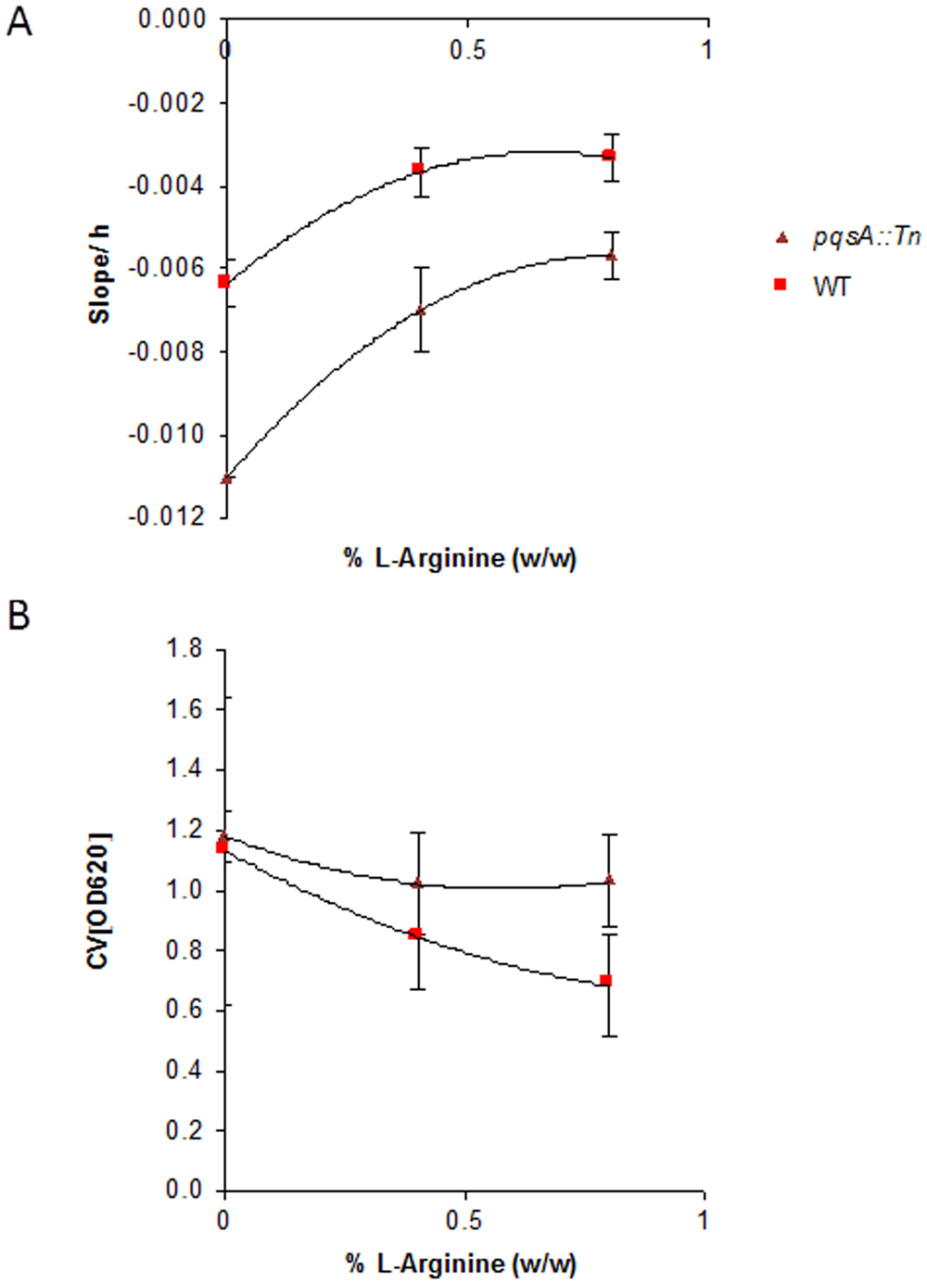
Impact of L-arginine (n=3) (**A**) on the cell index slope measured by single-frequency impedance spectroscopy over 41 h and (**B**) on the crystal violet staining intensity of PA14 wt and the *pqsA::Tn* mutant strains. The crystal violet background of the medium was subtracted. n indicates the number of technical replicates.

 Because the decline of the single-frequency impedance curve following the maximum after ca. 30–35 h coincided with the period of biofilm growth (yellow bar in Fig. 2A), and as it captured the effect of an added pellicle biofilm, the slope of the linear phase of the declining cell index, determined by fitting the data to a local linear model, was used as a parameter for pellicle biofilm formation. The decline of the cell index became less steep upon the addition of L-arginine in a concentration dependent manner; the effect occurred in both PA14 wt as well as the *pqsA::Tn* mutant (Fig. 4A, Table S2 and Figure S3). Furthermore, the biofilm formation started earlier with *pqsA::Tn* (28.4 h ± 0) than with the wt (30.5 h ± 0.7). Also, 0.4% and 0.8% (w/w) L-arginine shortened the induction time for the biofilm formation of *pqsA::Tn* 29.2 h ± 1.5, 26.7 h ± 0.7 and the wt 25.0 h ± 0.7, 24.2 h ± 0.7, respectively. In parallel, the biofilm growth under these conditions was monitored by crystal violet staining after 43 hours as a reference, showing reduced intensities for both strains upon addition of L-arginine, as expected (Fig. 4B). For the end point measurement with crystal violet, maximal fold changes of 0.40 and 0.13 in OD_620_ values with the wt and the *pqsA::Tn* mutant were measured with maximal relative standard deviations of 46% and 17%, respectively. Single-frequency impedance measurements showed fold changes of 0.48 and 0.47 in slope for the two strains, respectively, with corresponding maximal relative standard deviations of 17% and 14%. Thus, the higher dynamic range and the smaller standard deviation of the impedance measurements suggest that the method may be better suited to track subtle changes in biofilm growth than crystal violet measurements. In fact, recording multiple time points by the CV method requires is experimentally tedious, associated with large error bars between measurements, and the frequent removal of plates from the incubator constitutes a perturbation of microbial growth (Figure S4).

**Figure 5:**
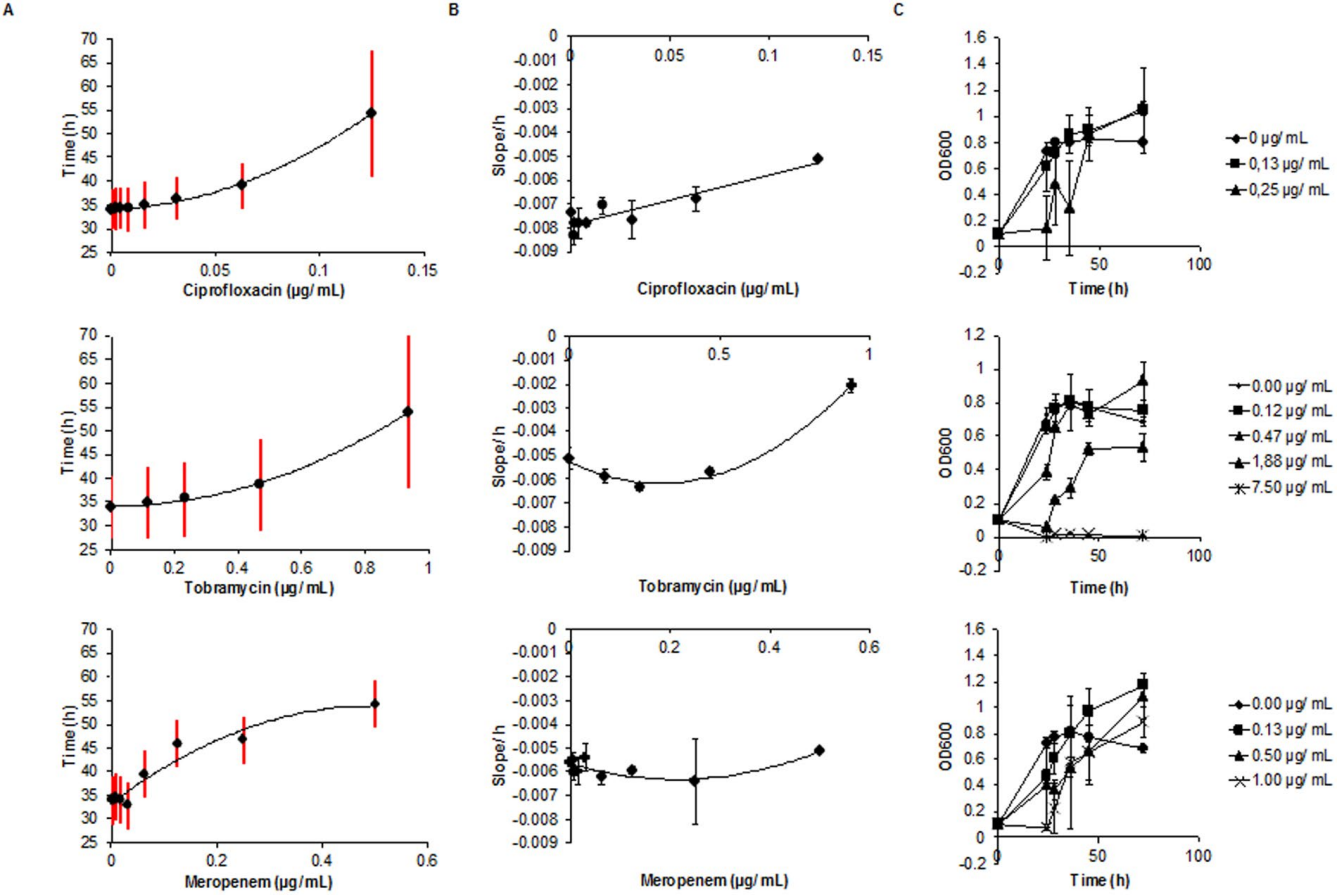
Impact of ciprofloxacin, tobramycin, and meropenem on biofilm formation of the PA14 wt strain over 70 h. (**A**) Impact of antibiotics on the time period of biofilm formation. The red vertical bars cover the time period of linear decline of the cell index value at a given antibiotic concentration, as determined by the fitting model. The lower end of the red bar marks the onset of CI decline, the upper end marks the end of the decline (n = 3 or 4). (**B**) The slope of the cell index is plotted against the antibiotic concentration (n = 3 or 4). (**C**) Growth curves of PA14 at various antibiotic concentrations over time (n = 4). The starting OD600 at t = 0 was 0.1. n indicates the number of technical replicates.

The method has also been used to study the effect of antibiotics on biofilm formation of the PA14 wt over 70 h. For this purpose, ciprofloxacin, tobramycin and meropenem have been tested, as they represent current standard treatments of *P. aeruginosa* infections. All three antibiotics changed the impedance curves in a concentration-dependent manner, indicating effects on biofilm formation (Fig. 5, Table S3, Figures S5 and S7). Interestingly, single-frequency impedance spectroscopy could monitor effects at antibiotic concentrations that did not impact planktonic growth in the same period of time. For example, the addition of 0.125 µg/ml ciprofloxacin clearly changed the impedance curve, although growth, determined by OD measurements of bacterial cultures in the presence of antibiotics, was not impaired at this concentration (Fig. 5C). At a concentration of 0.25 µg/ml, steady biofilm formation was not observed anymore, but planktonic growth was still possible. The effect on impedance was quantified by three parameters at varying antibiotic concentrations: The first parameter is the time of onset of a linear decrease of the cell index (Fig. 5A). The second parameter is the duration of a linear decrease of the cell index (Fig. 5A). The third parameter is the cell index slope during that time period (Fig. 5B). As outlined above, both parameters were determined by a linear trendline that was fitted to the data (Figures S5 and S7). For ciprofloxacin and tobramycin, a less steep decline could be observed at increasing concentrations, indicating an impaired biofilm formation. For meropenem on the other hand, the cell index slope was rather constant. Especially at the highest tested concentrations, all antibiotics led to later onsets of linear declines of impedance; at the same time, the period of linear decline was longer compared to untreated samples, indicating a delayed and attenuated biofilm formation. When antibiotic concentrations are sufficient to inhibit bacterial growth, changes in impedance diminish, and a constant curve is obtained (see for example Scheme S5, Tobramycin concentrations from 7.5 to 30 µg/ml).

## Discussion

The single-frequency impedance of microbial broth solutions is modulated by a variety of biological, chemical and electrochemical processes. As these processes are dynamic along the bacterial life cycle, the deconvolution of the main contributors to impedance is a subject of current research^59, 61^. By following the single-frequency impedance of *P. aeruginosa* PA14 over a time period of 72 h, we observed curve shapes that were featured by a maximum at ca. 30–35 h followed by a period of decline of ca. 10 h. These features could be abrogated by a *pelA::Tn* mutant that was not able of forming a biofilm, although it was not impaired in growth, which implies that the impedance curve is indeed shaped by biofilm formation. Bruchmann *et al*. reported a continuous rise of impedance over days at slightly higher starting OD’s (0.5 vs. 0.1 in our study) of *P. aeruginosa* cultures using a microfluidic device^60^, implying that the dynamics of the impedance signal is strongly influenced by the instrumental setup, i.e. by flow vs. static biofilm growth conditions. One important difference is that pellicle formation is hampered by the flow. This suggests that the pellicle may account for differences in signal shape between the studies. In our study, the effect of the pellicle biofilm was separated from that of the liquid broth by a transplant experiment, demonstrating that the pellicle alone changed single-frequency impedance. This effect cannot be explained by the addition of an immiscible layer on top of the LB medium, as the addition of paraffin, an inert material with low dielectric constant, led to unchanged slopes of the cell index. The finding highlights the pellicle biofilm formed at the air-liquid interface as another contributing factor to impedance. Ward *et al*. recently noted the pellicle effect as well when putting the electrode in contact with the liquid air interface^61^. The electrode in the present study is located at the bottom of the well distant from the pellicle, demonstrating that the pellicle can exert a remote effect at a distance of ca. 0.8 cm to the electron surface. We suggest that the pellicle may increase the capacitance of the material, thereby contributing to an overall decline of impedance. However, the molecular mechanism behind the effect remains unclear at this stage. This suggestion is in line with the fact that the linear decrease of the impedance curve coincides with the growth period of the pellicle, as monitored by crystal violet staining. The overall shape of the single-frequency impedance curve over 73 h is complex and influenced by various electrochemical mediators and processes along the growth phase of *P. aeruginosa*. Therefore, we do not imply that the cell index at any time is specific for biofilm formation. However, the comparison between biofilm formers and the respective isogenic controls cultivated under identical conditions suggests that the signal in a specific time period, which coincides with the formation of a pellicle biofilm, indeed reflects biofilm growth. We therefore suggest the length and the slope of the linear part of the cell index as simple descriptors of biofilm formation in *P. aeruginosa*.

In our studies with adherent eukaryotic cells, CI values up to +5 were observed (unpublished data). In contrast, the CI values detected for *P. aeruginosa* biofilms in this study were about 20fold smaller. As eukaryotic cells are larger and adhere to the electrode surface in dense (mono)layers, their impact on the resistance to impedance is a lot larger than that of *Pseudomonas* cells, whose attachment to the electrode surface was limited.

Using L-arginine as a suppressor of biofilm formation, a head-to-head comparison with crystal violet staining demonstrated that quantification by impedance curves was associated with higher dynamic ranges and smaller standard deviations than the notoriously variable crystal violet staining method, thereby enabling to disclose also subtle perturbances of a biofilm. This was further explored by monitoring the effects of three standard antibiotics on single-frequency impedance and its descriptors slope, onset and duration of linear decline. It is well-documented that antibiotics induce significant alterations of gene expression even at low doses^65–68^. We observed that sublethal concentrations that did not impair bacterial growth also altered the impedance of *P. aeruginosa* PA14 cultures, and that the substance effects were different. At higher concentrations, all antibiotics delayed the onset and extended the period of linear curve decline, which we interpret as a consequence of impaired biofilm formation.

In summary, single-frequency impedance spectroscopy with a commercially available instrument has been applied to characterize the growth dynamics of biofilm-forming *P. aeruginosa* strains in a label-free manner. The technique revealed distinct phases of biofilm formation and characterized the effects of antibiotics and other biofilm-modulating compounds with a precision that was superior to crystal violet staining. Using mutant strains and transplant experiments, we demonstrated that the pellicle biofilm formed at the air-liquid interface is an important contributor to impedance.

As the measurements are done in 96 well plates in an automated fashion, and as a 384 well plate format is supported by the instrument, single-frequency impedance spectroscopy may find utility for the medium throughput screening of compound libraries to discover modulators of biofilm formation. Future work will be devoted to extending the method to other biofilm-forming pathogens, and to gaining an enhanced understanding of impedance curve dynamics, for example to disclose the molecular processes that lead to the curve maximum. More detailed descriptor functions that also capture the earlier part of impedance curves may be conceived in order to obtain compound signatures that reflect a specific mode of action. While such an application of single-frequency impedance spectroscopy has been reported for eukaryotic cells^69, 70^, the current work suggests that it is also viable for biofilm-forming bacteria. A diagnostic utility for clinical purposes may be derived following miniaturization of the assays to a low-cost chip format, as demonstrated for other applications of impedance microbiology.

## Material and Methods

### Strains and cultivation conditions

The reference strain *Pseudomonas aeruginosa* PA14 (DSM 50071) and two mutant strains with a transposon insertion within the *pqsA::Tn* or the *pelA::Tn* gene were applied in this study^10, 17, 71^. Both mutants have been shown to result in a low biofilm morphotype^16, 18^. The mutant strains were obtained from the PA14 transposon mutant library of the F.M. Ausubel lab. And the strains were stored in glycerol stocks at −70 °C. The pre-cultures (4 ml lysogeny broth (LB) medium) were incubated overnight at 37 °C and 180 rpm. Chemicals were obtained from commercial sources (ciprofloxacin hydrochloride from Biomedicals no. 02199020, tobramycin sulfate salt from Serva no. 36562.01, meropenem trihydate from Sigma Aldrich no. M2574, L-arginine from AppliChem no. A3709). The compounds were diluted with LB media and distributed to the test plate just before the incubation with bacteria. The concentration ranges for the antibiotics were selected according to MIC values reported on the EUCAST website, while literature data were adapted for arginine concentrations^72^.

### Biofilm growth assay

The cultivation of static biofilms at the bottom of 96 well microtiter plates was performed as previously described^33^. Overnight cultures were adjusted with LB to an OD600 of 0.02. From these cultures, 100 µl were transferred into a half-area, μclear microtiter plate (Greiner Bio-One), covered with an air-permeable foil and incubated at 37 °C in an incubator with humid atmosphere for 24 h, 48 h and 72 h. To stain the bacteria, 60 µl of staining solution containing Syto9 and propidium iodide with final concentrations of 2.1 µM and 12.5 µM, respectively (LIVE/DEAD BacLight Bacterial Viability kit, Molecular Probes/Life Technologies), was added per well. Stacks of the biofilms were acquired with an automated confocal laser-scanning microscope (TCS SP8, Leica Microsystems) equipped with an x40/1.10 water objective and a z-step size of 3 μm between the image layers. The acquired image stacks were analysed with a customized solution from the software developer XD (Definiens) to calculate biofilm-relevant parameters and visualized with the software IMARIS (Bitplane).

### Crystal violet staining

To quantify biofilm formation, a 96 well plate was inoculated from a pre-culture^73^. LB start cultures were prepared with an OD600 of 0.1 (200 µl/well). To measure the attached ring of the pellicle biofilm, cultures were withdrawn by suction, and the wells were washed with 200 µl H_2_O to remove media and unattached cell material. To stain the biofilm, 150 µl crystal violet staining solution (0.1% m/v in H_2_O) was added per well and incubated for 30 min at room temperature. The staining solution was removed, and the wells were washed with 200 µl H_2_O. After the plates were dried at room temperature, 200 µl ethanol (95%) was added to each well to destain the pellicle biofilm ring for 30 min at room temperature. 125 µl of the violet ethanol solution was transferred into a fresh 96-well plate, and the absorbance was measured at 550, 580 and 620 nm.

### Single-frequency impedance measurement

Before inoculation with pre-cultures, the background impedance of the culture media was measured from 100 µl LB medium per well in an E-plate 96 (ACEA, USA) with the xCELLigence RTCA SP (single plate) instrument (ACEA, USA). The analyzer with the E-plate was placed in a 37 °C incubator with 5% CO_2_. For each well, the background was subtracted from the measurement points by setting the cell index to zero at t = 0. Thereby, the cell index is a self-calibrated value. After pausing the system, the E-plate was reinstalled in the tissue culture incubator after inoculating of the wells with 200 µl of a LB-diluted overnight culture (start OD_600_ of 0.1). In a 5 minute interval for each well the single-frequency impedance signal was monitored for at least 41 h with default settings. The xCELLigence instrument displays the impedance as a standardized dimensionless parameter termed cell index (CI). Because the change in the electrical impedance at a certain frequency is divided by the nominal impedance value, the CI represents a relative value. The CI at a given time point *t* (CI(*t*)) is calculated as follows (Eq. 1).

1 $${CI}({t})=\,[{R}({f}n,{t})-{R}({f}n,{t}0)]/{Zn}$$

where *ƒn* is the frequency at which the impedance measurement is carried out; *R*(*ƒn*, *t*) is the measured impedance at frequency *ƒn* at time point *t; R*(*ƒn*, *t*0) is the measured impedance at frequency *ƒn* at time point *t*0 (usually *t*0 is the time when the background is measured) and *Zn* is the corresponding nominal impedance value of *ƒn*. All experiments have been performed at a frequency of 10 kHz and a nominal impedance value of 15 Ohm.

The observed CI values fell into the measurement range of the XCellingence instrument. The lower limit of CI for a reproducible measurement is 0.01–0.02, translating to an impedance change of 0.15 ohm–0.3 ohm. Whilst the impedance analyzer could certainly reproducibly resolve 0.15–0.3 ohm impedance changes, the RTCA system measurement could be affected by other factors such electrical contact resistance between the E-Plate and E-Plate station and other parts of measurement system. The upper limit of CI is experted to be >100, since the system could reproducibly measure the impedance up to many thousands of ohms, or even higher^74^.

The electrical potential at the electrodes is about 10 mV. The Alternating Current (AC) voltage is from an impedance analyzer where the voltage signal is digitally synthesized to a voltage amplitude (about 20 mV) and sinusoidal waveform of several variable frequencies (e.g. 10 kHz, 25 kHz, 50 kHz). There is no reference electrode in the system. The AC voltage signal from the analyzer is applied to the electrodes through electronic switching circuits. The system measured AC electrical current by monitoring the voltage drop across of an internal resistance. The impedance is calculated from the division of the voltage by the current^74^. All experiments in the study have been conducted at a frequency of 10 kHz. The open circuit potential remains constant during the experiment. The change in the electrical current going through the system leads to the derivation of the change in the monitored impedance.

The gold electrodes are of interdigitated, circle-on-line electrode geometry. The circle is of 90 μm diameter. The separation between the neighboring electrode lines is 20 μm. The electrodes are fabricated on glass substrate with lithographic techniques^75^.

The equivalent circuit of impedance (*Z*) can be described as a serial combination of the resistance (*Z*_*R*_ = *R*) and reactance (*Z*_*C*_ = 1/*jwc*) (Eq. 2).

2 $$Z={Z}_{R}+{Z}_{C}=R+1/j\omega C$$

For identification of the linear range of cell index decay, reflecting the biofilm growth period, a 5 h sliding window was moved in 1 hour steps along the time axis, and a local linear model was fitted. Only data from 20 h onwards was considered, based on empirical data on the lag phase of *P. aeruginosa* biofilm growth. Next, local linear models were removed if r^2^ was below the cut-off of 0.97 (Figure S1). In the presence of tobramycin, the r^2^ was adapted to 0.89 based on a higher noise ratio at a concentration of 0.94 µg/ml. This however did not lead to an inaccurate measurement, given the calculated variances of slopes of sliding windows in the presence of tobramycin did not increase (Figure S7). The range of biofilm growth was defined as beginning from the start of the first to the end of the last of a series of consecutive sliding windows fulfilling the defined criteria, while allowing for a maximum of one local linear model with r^2^ below the cutoff.

To mimic the presence of pellicle biofilm in a point measurement, paraffin, which is a hydrophobic, electrically inactive compound, was added in different amounts (10–100 µL) (n = 4) to separate wells that contained LB medium. Paraffin is immiscible with water and floats on top of the medium due to its lower density. In a second set of experiments, biofilm grown in a 96 well plate in LB medium for 72 h was transplanted with a metal loop into four wells filled with LB medium. To transplant the pellicle biofilm, it was first carefully loosened by adding 30 µl PBS under the pellicle biofilm structure with a pipette. In control wells, varying amounts of PBS buffer (10, 20, 40, and 80 µl) (n = 1) were added to capture the inpact of PBS on single-frequency impedance. CI values were compared to the LB medium (n = 8). In both sets of experiments, the xCELLigence system was paused before addition.

## Electronic supplementary material

Supplementary Information
