# Supplementary material for: Use of Single-Frequency Impedance Spectroscopy to Characterize the Growth Dynamics of Biofilm Formation in Pseudomonas aeruginosa
Source: Sci Rep. 2017 Jul 12;7:5223. doi: 10.1038/s41598-017-05273-5 (PMC5507860; doi:10.1038/s41598-017-05273-5)
Supplement: Supplementary file 1 — Supplementary Information [file 41598_2017_5273_MOESM1_ESM.doc]

SUPPORTING INFORMATION

**Use of Impedance Spectroscopy to Characterize the Growth Dynamics of Biofilm Formation in *Pseudomonas aeruginosa***

Jozef B.J.H. van Duuren, Mathias Müsken, Bianka Karge, Jürgen Tomasch, Christoph Wittmann, Susanne Häussler, Mark Brönstrup

Table of Content

[Figure S1. Identification of parameters reflecting biofilm growth. 2](#__RefHeading___Toc483413895)

[Table S1. Impedance curve parameters for *P. aeruginosa* mutants. 3](#__RefHeading___Toc483413896)

[Figure S2. Impedance curves and fitted parameters for *P. aeruginosa* PA14 wt and the pqsA and pelA mutants. 5](#__RefHeading___Toc483413897)

[Table S2. Effect of arginine on impedance curve parameters. 12](#__RefHeading___Toc483413898)

[Figure S3. Effect of arginine on impedance curves. 13](#__RefHeading___Toc483413899)

[Figure S4. Biofilm growth recorded by crystal violet staining across multiple time points. 15](#__RefHeading___Toc483413900)

[Table S3. Effect of antibiotics on impedance curve parameters. 16](#__RefHeading___Toc483413901)

[Figure S5. Effect of antibiotics on impedance curves. 20](#__RefHeading___Toc483413902)

[Figure S6. Schematic overview of the transplant experiments with *P. aeruginosa* biofilms. 53](#__RefHeading___Toc483413903)

[Figure S7. Boxplot representation of the variance of measured slopes in the presence of antibiotics. 54](#__RefHeading___Toc483413904)

Figure S1. Identification of parameters reflecting biofilm growth. Two examples are given. Upper panels show the full measurement period, while lower panels show a close-up of the period of active growth for (A) *P. aeruginosa* WT grown without antibiotics and (B) *P. aeruginosa* grown with 0.064 μg/ml ciprofloxacin. The black line marks the starting point of the sliding window for fitting linear models that is set to 20h in all cases. The blue lines mark the identified start- and end-points of the linear period of decline of the cell index. The red line shows the fitted model.

Table S1. Impedance curve parameters for *P. aeruginosa* mutants. Starting points, endpoints and slopes of the linear period of decline of the cell index of PA 14 wt, and the *pqsA* and *pelA* mutants (24 technical replicates for each mutant), and the LB medium control (8 technical replicates) by curve fitting. Entries in red indicate that a curve fitting could not be achieved.

| **ID** | **Strain** | **Min (h)** | | **Max (h)** | | **Slope/ h** |
| --- | --- | --- | --- | --- | --- | --- |
| A8 | *P. aeruginosa*-WT | 31.17 | 42.68 | | -0.006 | |
| A9 | *P. aeruginosa*-WT | 32.42 | 41.43 | | -0.008 | |
| A10 | *P. aeruginosa*-WT | 32.42 | 42.68 | | -0.007 | |
| B8 | *P. aeruginosa*-WT | 32.42 | 43.93 | | -0.006 | |
| B9 | *P. aeruginosa*-WT | 33.67 | 46.43 | | -0.006 | |
| B10 | *P. aeruginosa*-WT | 32.42 | 43.93 | | -0.006 | |
| C8 | *P. aeruginosa*-WT | 32.42 | 43.93 | | -0.006 | |
| C9 | *P. aeruginosa*-WT | 33.67 | 46.43 | | -0.006 | |
| C10 | *P. aeruginosa*-WT | 33.67 | 46.43 | | -0.006 | |
| D8 | *P. aeruginosa*-WT | 32.42 | 43.93 | | -0.005 | |
| D9 | *P. aeruginosa*-WT | 33.67 | 46.43 | | -0.006 | |
| D10 | *P. aeruginosa*-WT | 32.42 | 43.93 | | -0.006 | |
| E8 | *P. aeruginosa*-WT | 32.42 | 43.93 | | -0.006 | |
| E9 | *P. aeruginosa*-WT | 32.42 | 45.18 | | -0.006 | |
| E10 | *P. aeruginosa*-WT | 33.67 | 46.43 | | -0.006 | |
| F8 | *P. aeruginosa*-WT | 32.42 | 45.18 | | -0.005 | |
| F9 | *P. aeruginosa*-WT | 33.67 | 45.18 | | -0.006 | |
| F10 | *P. aeruginosa*-WT | 33.67 | 45.18 | | -0.006 | |
| G8 | *P. aeruginosa*-WT | 32.42 | 43.93 | | -0.006 | |
| G9 | *P. aeruginosa*-WT | 33.67 | 46.43 | | -0.006 | |
| G10 | *P. aeruginosa*-WT | 33.67 | 46.43 | | -0.006 | |
| H8 | *P. aeruginosa*-WT | 32.42 | 48.94 | | -0.005 | |
| H9 | *P. aeruginosa*-WT | 33.67 | 43.93 | | -0.006 | |
| H10 | *P. aeruginosa*-WT | 32.42 | 43.93 | | -0.006 | |
| A5 | *P. aeruginosa-*Δ*pqsA* | 29.92 | 48.94 | | -0.006 | |
| A6 | *P. aeruginosa-*Δ*pqsA* | 29.92 | 48.94 | | -0.005 | |
| A7 | *P. aeruginosa-*Δ*pqsA* | 31.17 | 53.94 | | -0.005 | |
| B5 | *P. aeruginosa-*Δ*pqsA* | 31.17 | 45.18 | | -0.006 | |
| B6 | *P. aeruginosa-*Δ*pqsA* | 32.42 | 47.69 | | -0.005 | |
| B7 | *P. aeruginosa-*Δ*pqsA* | 32.42 | 47.69 | | -0.006 | |
| C5 | *P. aeruginosa-*Δ*pqsA* | 31.17 | 43.93 | | -0.006 | |
| C6 | *P. aeruginosa-*Δ*pqsA* | 31.17 | 43.93 | | -0.006 | |
| C7 | *P. aeruginosa-*Δ*pqsA* | 31.17 | 47.69 | | -0.006 | |
| D5 | *P. aeruginosa-*Δ*pqsA* | 31.17 | 50.19 | | -0.005 | |
| D6 | *P. aeruginosa-*Δ*pqsA* | 31.17 | 43.93 | | -0.006 | |
| D7 | *P. aeruginosa-*Δ*pqsA* | 31.17 | 46.43 | | -0.006 | |
| E5 | *P. aeruginosa-*Δ*pqsA* | 31.17 | 52.69 | | -0.005 | |
| E6 | *P. aeruginosa-*Δ*pqsA* | 32.42 | 45.18 | | -0.006 | |
| E7 | *P. aeruginosa-*Δ*pqsA* | 31.17 | 50.19 | | -0.005 | |
| F5 | *P. aeruginosa-*Δ*pqsA* | 31.17 | 46.43 | | -0.005 | |
| F6 | *P. aeruginosa-*Δ*pqsA* | 31.17 | 43.93 | | -0.005 | |
| F7 | *P. aeruginosa-*Δ*pqsA* | 31.17 | 45.18 | | -0.006 | |
| G5 | *P. aeruginosa-*Δ*pqsA* | 31.17 | 51.44 | | -0.005 | |
| G6 | *P. aeruginosa-*Δ*pqsA* | 31.17 | 45.18 | | -0.005 | |
| G7 | *P. aeruginosa-*Δ*pqsA* | 31.17 | 46.43 | | -0.005 | |
| H5 | *P. aeruginosa-*Δ*pqsA* | 31.17 | 47.69 | | -0.005 | |
| H6 | *P. aeruginosa-*Δ*pqsA* | 29.92 | 48.94 | | -0.004 | |
| H7 | *P. aeruginosa-*Δ*pqsA* | 31.17 | 50.19 | | -0.005 | |
| A2 | *P. aeruginosa-*Δ*pelA* | ND | ND | | ND | |
| A3 | *P. aeruginosa-*Δ*pelA* | ND | ND | | ND | |
| A4 | *P. aeruginosa-*Δ*pelA* | ND | ND | | ND | |
| B2 | *P. aeruginosa-*Δ*pelA* | ND | ND | | ND | |
| B3 | *P. aeruginosa-*Δ*pelA* | ND | ND | | ND | |
| B4 | *P. aeruginosa-*Δ*pelA* | ND | ND | | ND | |
| C2 | *P. aeruginosa-*Δ*pelA* | ND | ND | | ND | |
| C3 | *P. aeruginosa-*Δ*pelA* | ND | ND | | ND | |
| C4 | *P. aeruginosa-*Δ*pelA* | ND | ND | | ND | |
| D2 | *P. aeruginosa-*Δ*pelA* | ND | ND | | ND | |
| D3 | *P. aeruginosa-*Δ*pelA* | ND | ND | | ND | |
| D4 | *P. aeruginosa-*Δ*pelA* | ND | ND | | ND | |
| E2 | *P. aeruginosa-*Δ*pelA* | ND | ND | | ND | |
| E3 | *P. aeruginosa-*Δ*pelA* | ND | ND | | ND | |
| E4 | *P. aeruginosa-*Δ*pelA* | ND | ND | | ND | |
| F2 | *P. aeruginosa-*Δ*pelA* | ND | ND | | ND | |
| F3 | *P. aeruginosa-*Δ*pelA* | ND | ND | | ND | |
| F4 | *P. aeruginosa-*Δ*pelA* | ND | ND | | ND | |
| G2 | *P. aeruginosa-*Δ*pelA* | ND | ND | | ND | |
| G3 | *P. aeruginosa-*Δ*pelA* | ND | ND | | ND | |
| G4 | *P. aeruginosa-*Δ*pelA* | ND | ND | | ND | |
| H2 | *P. aeruginosa-*Δ*pelA* | ND | ND | | ND | |
| H3 | *P. aeruginosa-*Δ*pelA* | ND | ND | | ND | |
| H4 | *P. aeruginosa-*Δ*pelA* | ND | ND | | ND | |
| A1 | LB-Medium | ND | ND | | ND | |
| B1 | LB-Medium | ND | ND | | ND | |
| C1 | LB-Medium | ND | ND | | ND | |
| D1 | LB-Medium | ND | ND | | ND | |
| E1 | LB-Medium | ND | ND | | ND | |
| F1 | LB-Medium | ND | ND | | ND | |
| G1 | LB-Medium | ND | ND | | ND | |
| H1 | LB-Medium | ND | ND | | ND | |

# Figure S2. Impedance curves and fitted parameters for *P. aeruginosa* PA14 wt and the pqsA and pelA mutants.

The Identifiers correspond to those found in Table S1.


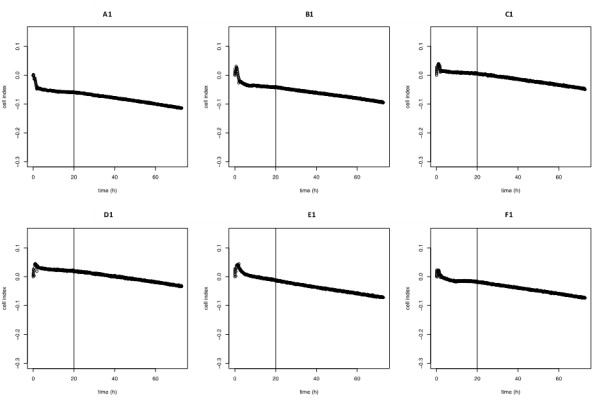


Table S2. Effect of arginine on impedance curve parameters. Parameters were obtained for PA 14 WT and Δ*pqsA* mutants in the presence of 0%, 0.4% and 0.8% (w/w) *L*-arginine over 41h (3 technical replicates).

| **ID** | **Strain** | **Min (h)** | **Max (h)** | **Slope/ h** |
| --- | --- | --- | --- | --- |
| WT | *P. aeruginosa* | 29.63 | 41.14 | -0.007 |
| WT.1 | *P. aeruginosa* | 30.88 | 41.14 | -0.006 |
| WT.2 | *P. aeruginosa* | 30.88 | 41.14 | -0.006 |
| WT.0.4.ARG | *P. aeruginosa* | 28.37 | 41.14 | -0.004 |
| WT.0.4.ARG.1 | *P. aeruginosa* | 30.88 | 41.14 | -0.004 |
| WT.0.4.ARG.2 | *P. aeruginosa* | 28.37 | 41.14 | -0.003 |
| WT.0.8.ARG | *P. aeruginosa* | 27.12 | 41.14 | -0.004 |
| WT.0.8.ARG.1 | *P. aeruginosa* | 27.12 | 41.14 | -0.003 |
| WT.0.8.ARG.2 | *P. aeruginosa* | 25.87 | 41.14 | -0.003 |
| ∆*pqsA* | *P. aeruginosa* | 28.37 | 41.14 | -0.011 |
| ∆*pqsA*.1 | *P. aeruginosa* | 28.37 | 41.14 | -0.011 |
| ∆*pqsA*.2. | *P. aeruginosa* | 28.37 | 41.14 | -0.011 |
| ∆*pqsA*.0.4.ARG | *P. aeruginosa* | 25.87 | 41.14 | -0.006 |
| ∆*pqsA*.0.4.ARG.1 | *P. aeruginosa* | 24.62 | 41.14 | -0.007 |
| ∆*pqsA*.0.4.ARG.2 | *P. aeruginosa* | 24.62 | 41.14 | -0.008 |
| ∆*pqsA*.0.8.ARG | *P. aeruginosa* | 24.62 | 41.14 | -0.006 |
| ∆*pqsA*.0.8.ARG.1 | *P. aeruginosa* | 23.36 | 41.14 | -0.005 |
| ∆*pqsA*.0.8.ARG.2 | *P. aeruginosa* | 24.62 | 41.14 | -0.006 |

Figure S3. Effect of arginine on impedance curves. Impedance curves and fitted parameters are shown for PA 14 WT and Δ*pqsA* mutants in the presence of 0%, 0.4% and 0.8% (w/w) *L*-arginine over 41h (3 technical replicates).

Figure S4. Biofilm growth recorded by crystal violet staining across multiple time points. CV staining intensities of PA14 pqsA::Tn (red curve) and of PA14 pelA::Tn (blue curve) over 17 time points. At each time point, the CV staining intensities were measured in 24 different wells of a microtiter plate and averaged.

Table S3. Effect of antibiotics on impedance curve parameters. Parameters were obtained for PA 14 WT in the presence of ciprofloxacin (cipro) (0-0.25 μg/mL), tobramycin (tobra) (0-30 μg/mL), or meropenem (mero) (0-1 μg/mL) over 70h. Entries in red indicate that a curve fitting could not be achieved.

| **ID** | **Strain** | **Substance** | **Concentration (µg/mL)** | **Min (h)** | **Max (h)** | **Slope/ h** |
| --- | --- | --- | --- | --- | --- | --- |
| C1 | *P. aeruginosa* | cipro | 0 | 28.61 | 36.36 | -0.007 |
| C2 | *P. aeruginosa* | cipro | 0 | 29.86 | 37.61 | -0.007 |
| C3 | *P. aeruginosa* | cipro | 0 | 29.86 | 38.86 | -0.007 |
| C4 | *P. aeruginosa* | cipro | 0 | 29.86 | 37.61 | -0.007 |
| C5 | *P. aeruginosa* | cipro | 0 | 29.86 | 38.86 | -0.007 |
| C6 | *P. aeruginosa* | cipro | 0 | 29.86 | 37.61 | -0.008 |
| C7 | *P. aeruginosa* | cipro | 0 | 29.86 | 38.86 | -0.008 |
| C8 | *P. aeruginosa* | cipro | 0 | 29.86 | 40.12 | -0.008 |
| C9 | *P. aeruginosa* | cipro | 0.001 | 28.61 | 37.61 | -0.009 |
| C10 | *P. aeruginosa* | cipro | 0.001 | 29.86 | 37.61 | -0.009 |
| C11 | *P. aeruginosa* | cipro | 0.001 | 29.86 | 38.86 | -0.008 |
| C12 | *P. aeruginosa* | cipro | 0.001 | 31.11 | 38.86 | -0.008 |
| C13 | *P. aeruginosa* | cipro | 0.002 | 28.61 | 37.61 | -0.008 |
| C14 | *P. aeruginosa* | cipro | 0.002 | 29.86 | 38.86 | -0.008 |
| C15 | *P. aeruginosa* | cipro | 0.002 | 29.86 | 40.12 | -0.008 |
| C16 | *P. aeruginosa* | cipro | 0.002 | 29.86 | 38.86 | -0.008 |
| C17 | *P. aeruginosa* | cipro | 0.004 | 29.86 | 38.86 | -0.007 |
| C18 | *P. aeruginosa* | cipro | 0.004 | 29.86 | 38.86 | -0.008 |
| C19 | *P. aeruginosa* | cipro | 0.004 | 29.86 | 40.12 | -0.008 |
| C20 | *P. aeruginosa* | cipro | 0.004 | 29.86 | 37.61 | -0.008 |
| C21 | *P. aeruginosa* | cipro | 0.008 | 29.86 | 38.86 | -0.008 |
| C22 | *P. aeruginosa* | cipro | 0.008 | 28.61 | 38.86 | -0.008 |
| C23 | *P. aeruginosa* | cipro | 0.008 | 29.86 | 38.86 | -0.008 |
| C24 | *P. aeruginosa* | cipro | 0.016 | 29.86 | 37.61 | -0.007 |
| C25 | *P. aeruginosa* | cipro | 0.016 | 31.11 | 41.37 | -0.007 |
| C26 | *P. aeruginosa* | cipro | 0.016 | 29.86 | 40.12 | -0.007 |
| C27 | *P. aeruginosa* | cipro | 0.016 | 29.86 | 40.12 | -0.007 |
| C28 | *P. aeruginosa* | cipro | 0.032 | 31.11 | 40.12 | -0.007 |
| C29 | *P. aeruginosa* | cipro | 0.032 | 32.36 | 41.37 | -0.008 |
| C30 | *P. aeruginosa* | cipro | 0.032 | 31.11 | 41.37 | -0.009 |
| C31 | *P. aeruginosa* | cipro | 0.032 | 32.36 | 41.37 | -0.008 |
| C32 | *P. aeruginosa* | cipro | 0.064 | 33.61 | 41.37 | -0.006 |
| C33 | *P. aeruginosa* | cipro | 0.064 | 34.86 | 45.12 | -0.007 |
| C34 | *P. aeruginosa* | cipro | 0.064 | 33.61 | 43.87 | -0.007 |
| C35 | *P. aeruginosa* | cipro | 0.064 | 34.86 | 45.12 | -0.006 |
| C36 | *P. aeruginosa* | cipro | 0.125 | NA | NA | NA |
| C37 | *P. aeruginosa* | cipro | 0.125 | 41.12 | 68.88 | -0.005 |
| C38 | *P. aeruginosa* | cipro | 0.125 | 41.12 | 67.63 | -0.005 |
| C39 | *P. aeruginosa* | cipro | 0.125 | 41.12 | 66.38 | -0.005 |
| C40 | *P. aeruginosa* | cipro | 0.25 | NA | NA | NA |
| C41 | *P. aeruginosa* | cipro | 0.25 | NA | NA | NA |
| C42 | *P. aeruginosa* | cipro | 0.25 | NA | NA | NA |
| C43 | *P. aeruginosa* | cipro | 0.25 | NA | NA | NA |

| **ID** | **Strain** | **Substance** | **Concentration (µg/mL)** | **Min (h)** | **Max (h)** | **Slope/ h** |
| --- | --- | --- | --- | --- | --- | --- |
| T1 | *P. aeruginosa* | tobra | 0 | 27.27 | 40.02 | -0.01 |
| T2 | *P. aeruginosa* | tobra | 0 | 27.27 | 41.27 | 0.00 |
| T3 | *P. aeruginosa* | tobra | 0 | 27.27 | 41.27 | 0.00 |
| T4 | *P. aeruginosa* | tobra | 0 | 27.27 | 40.02 | 0.00 |
| T5 | *P. aeruginosa* | tobra | 0 | 26.02 | 40.02 | -0.01 |
| T6 | *P. aeruginosa* | tobra | 0 | 28.52 | 40.02 | -0.01 |
| T7 | *P. aeruginosa* | tobra | 0 | 27.27 | 41.27 | -0.01 |
| T8 | *P. aeruginosa* | tobra | 0 | 27.27 | 40.02 | -0.01 |
| T9 | *P. aeruginosa* | tobra | 0.1172 | 27.27 | 42.52 | -0.01 |
| T10 | *P. aeruginosa* | tobra | 0.1172 | 27.27 | 42.52 | -0.01 |
| T11 | *P. aeruginosa* | tobra | 0.1172 | 27.27 | 42.52 | -0.01 |
| T12 | *P. aeruginosa* | tobra | 0.1172 | 27.27 | 41.27 | -0.01 |
| T13 | *P. aeruginosa* | tobra | 0.2344 | 28.52 | 43.77 | -0.01 |
| T14 | *P. aeruginosa* | tobra | 0.2344 | 28.52 | 43.77 | -0.01 |
| T15 | *P. aeruginosa* | tobra | 0.2344 | 27.27 | 42.52 | -0.01 |
| T16 | *P. aeruginosa* | tobra | 0.2344 | 27.27 | 43.77 | -0.01 |
| T17 | *P. aeruginosa* | tobra | 0.4688 | 42.27 | 70.03 | 0.00 |
| T18 | *P. aeruginosa* | tobra | 0.4688 | 28.52 | 48.78 | -0.01 |
| T19 | *P. aeruginosa* | tobra | 0.4688 | 28.52 | 47.53 | -0.01 |
| T20 | *P. aeruginosa* | tobra | 0.4688 | 29.77 | 48.78 | -0.01 |
| T21 | *P. aeruginosa* | tobra | 0.9375 | 37.27 | 70.03 | 0.00 |
| T22 | *P. aeruginosa* | tobra | 0.9375 | 41.02 | 70.03 | 0.00 |
| T23 | *P. aeruginosa* | tobra | 0.9375 | 33.52 | 70.03 | 0.00 |
| T24 | *P. aeruginosa* | tobra | 0.9375 | 39.77 | 70.03 | 0.00 |
| T25 | *P. aeruginosa* | tobra | 1.875 | NA | NA | NA |
| T26 | *P. aeruginosa* | tobra | 1.875 | NA | NA | NA |
| T27 | *P. aeruginosa* | tobra | 1.875 | NA | NA | NA |
| T28 | *P. aeruginosa* | tobra | 1.875 | NA | NA | NA |
| T29 | *P. aeruginosa* | tobra | 3.75 | NA | NA | NA |
| T30 | *P. aeruginosa* | tobra | 3.75 | NA | NA | NA |
| T31 | *P. aeruginosa* | tobra | 3.75 | NA | NA | NA |
| T32 | *P. aeruginosa* | tobra | 3.75 | NA | NA | NA |
| T33 | *P. aeruginosa* | tobra | 7.5 | NA | NA | NA |
| T34 | *P. aeruginosa* | tobra | 7.5 | NA | NA | NA |
| T35 | *P. aeruginosa* | tobra | 7.5 | NA | NA | NA |
| T36 | *P. aeruginosa* | tobra | 7.5 | NA | NA | NA |
| T37 | *P. aeruginosa* | tobra | 15 | NA | NA | NA |
| T38 | *P. aeruginosa* | tobra | 15 | NA | NA | NA |
| T39 | *P. aeruginosa* | tobra | 15 | NA | NA | NA |
| T40 | *P. aeruginosa* | tobra | 15 | NA | NA | NA |
| T41 | *P. aeruginosa* | tobra | 30 | NA | NA | NA |
| T42 | *P. aeruginosa* | tobra | 30 | NA | NA | NA |
| T43 | *P. aeruginosa* | tobra | 30 | NA | NA | NA |
| T44 | *P. aeruginosa* | tobra | 30 | NA | NA | NA |

| **ID** | **Strain** | **Substance** | **Concentration (µg/mL)** | **Min (h)** | **Max (h)** | **Slope/ h** |
| --- | --- | --- | --- | --- | --- | --- |
| M1 | *P. aeruginosa* | mero | 0 | 28.52 | 40.02 | -0.006 |
| M2 | *P. aeruginosa* | mero | 0 | 28.52 | 40.02 | -0.005 |
| M3 | *P. aeruginosa* | mero | 0 | 28.52 | 40.02 | -0.005 |
| M4 | *P. aeruginosa* | mero | 0 | 29.77 | 38.77 | -0.006 |
| M5 | *P. aeruginosa* | mero | 0 | 27.27 | 38.77 | -0.005 |
| M6 | *P. aeruginosa* | mero | 0 | 29.77 | 40.02 | -0.005 |
| M7 | *P. aeruginosa* | mero | 0 | 28.52 | 40.02 | -0.006 |
| M8 | *P. aeruginosa* | mero | 0 | 27.27 | 38.77 | -0.006 |
| M9 | *P. aeruginosa* | mero | 0.004 | 27.27 | 38.77 | -0.006 |
| M10 | *P. aeruginosa* | mero | 0.004 | 28.52 | 40.02 | -0.005 |
| M11 | *P. aeruginosa* | mero | 0.004 | 28.52 | 40.02 | -0.005 |
| M12 | *P. aeruginosa* | mero | 0.004 | 28.52 | 40.02 | -0.006 |
| M13 | *P. aeruginosa* | mero | 0.008 | 28.52 | 40.02 | -0.007 |
| M14 | *P. aeruginosa* | mero | 0.008 | 29.77 | 41.27 | -0.006 |
| M15 | *P. aeruginosa* | mero | 0.008 | 28.52 | 40.02 | -0.006 |
| M16 | *P. aeruginosa* | mero | 0.008 | 28.52 | 40.02 | -0.006 |
| M17 | *P. aeruginosa* | mero | 0.016 | 27.27 | 38.77 | -0.007 |
| M18 | *P. aeruginosa* | mero | 0.016 | 28.52 | 40.02 | -0.005 |
| M19 | *P. aeruginosa* | mero | 0.016 | 28.52 | 40.02 | -0.006 |
| M20 | *P. aeruginosa* | mero | 0.016 | 49.78 | 65.03 | -0.003 |
| M21 | *P. aeruginosa* | mero | 0.032 | 26.02 | 38.77 | -0.006 |
| M22 | *P. aeruginosa* | mero | 0.032 | 26.02 | 40.02 | -0.005 |
| M23 | *P. aeruginosa* | mero | 0.032 | 26.02 | 40.02 | -0.006 |
| M24 | *P. aeruginosa* | mero | 0.064 | 27.27 | 50.03 | -0.007 |
| M25 | *P. aeruginosa* | mero | 0.064 | 26.02 | 55.03 | -0.006 |
| M26 | *P. aeruginosa* | mero | 0.064 | 27.27 | 48.78 | -0.006 |
| M27 | *P. aeruginosa* | mero | 0.064 | 27.27 | 53.78 | -0.006 |
| M28 | *P. aeruginosa* | mero | 0.125 | 36.02 | 56.28 | -0.006 |
| M29 | *P. aeruginosa* | mero | 0.125 | 34.77 | 70.03 | -0.004 |
| M30 | *P. aeruginosa* | mero | 0.125 | 36.02 | 58.78 | -0.006 |
| M31 | *P. aeruginosa* | mero | 0.125 | 36.02 | 52.53 | -0.006 |
| M32 | *P. aeruginosa* | mero | 0.25 | 36.02 | 60.03 | -0.006 |
| M33 | *P. aeruginosa* | mero | 0.25 | 41.02 | 70.03 | -0.005 |
| M34 | *P. aeruginosa* | mero | 0.25 | 37.27 | 62.53 | -0.005 |
| M35 | *P. aeruginosa* | mero | 0.25 | 34.77 | 48.78 | -0.008 |
| M36 | *P. aeruginosa* | mero | 0.5 | NA | NA | NA |
| M37 | *P. aeruginosa* | mero | 0.5 | 41.02 | 68.78 | -0.005 |
| M38 | *P. aeruginosa* | mero | 0.5 | 41.02 | 67.53 | -0.005 |
| M39 | *P. aeruginosa* | mero | 0.5 | 41.02 | 66.28 | -0.005 |
| M40 | *P. aeruginosa* | mero | 1 | NA | NA | NA |
| M41 | *P. aeruginosa* | mero | 1 | NA | NA | NA |
| M42 | *P. aeruginosa* | mero | 1 | NA | NA | NA |
| M43 | *P. aeruginosa* | mero | 1 | NA | NA | NA |

Figure S5. Effect of antibiotics on impedance curves. Impedance curves and fitted parameters are shown for PA 14 WT in the presence of ciprofloxacin (cipro) (0-0.25 μg/mL), tobramycin (tobra) (0-30 μg/mL), or meropenem (mero) (0-1 μg/mL) over 70h.

Figure S6. Schematic overview of the transplant experiments with *P. aeruginosa* biofilms. (A) Biofilm grown in a 96 well microtiter plate in LB medium for 72 h (200 µl/well). (B) The biofilm was loosened by adding 30 µl PBS under the pellicle biofilm structure with a pipette. (C, D, E) Transplantation of the loosened biofilm with a metal loop into an Eplate well filled with LB medium. (F) The cell index of LB medium was first set to zero. (G) Subsequently, the xCELLigence system was paused before the addition of the biofilm. (H) Transplanted biofilm to mimic the presence of pellicle biofilm with a single frequency impedance spectroscopy measurement.

**
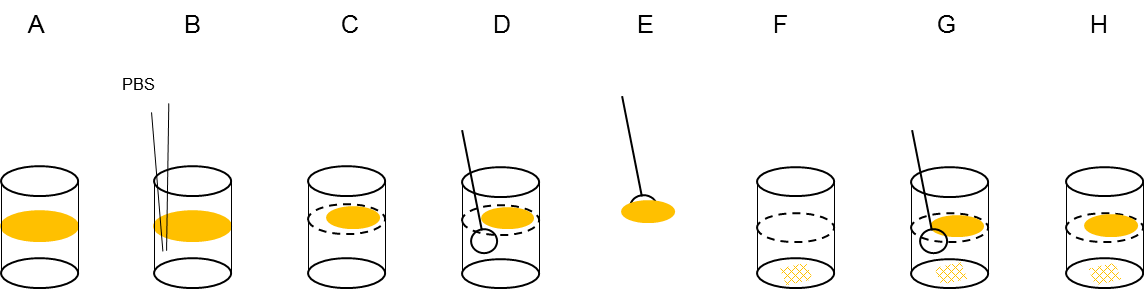
**

Figure S7. Boxplot representation of the variance of measured slopes in the presence of antibiotics. The entries in each well position are shown in Table S3. For each impedance curve, a series of slopes is obtained, as multiple sliding windows fulfill the condition of linear curve fitting. The variance of slope measurements is given in form of box-whisker plots.
